# Supplementary material for: Biosynthesis, characterization and anthelmintic activity of silver nanoparticles of Clerodendrum infortunatum isolate
Source: Sci Rep. 2023 May 7;13:7415. doi: 10.1038/s41598-023-34221-9 (PMC10164746; doi:10.1038/s41598-023-34221-9)
Supplement: Supplementary file 1 — Supplementary Table S1. [file 41598_2023_34221_MOESM1_ESM.docx]

**Supplementary Information**

**Biosynthesis, Characterization and Anthelmintic Activity of Silver Nanoparticles of *Clerodendrum* *infortunatum* Isolate**

Rima Majumdar, Pradip Kumar Kar^*^

| Test material |  |  | Concentration  (µg/ml PBS) | Paralysis (h) | Death (h) |
| --- | --- | --- | --- | --- | --- |
| Control |  |  | _ | _ | 72 ± 0.04 |
| CLE-aqueous extract |  |  | 25 | 3.96 ± 0.08 | 4.56 ± 0.09 |
|  |  |  | 50 | 3.84 ± 0.04 | 4.32 ± 0.08 |
|  |  |  | 75 | 3.43 ± 0.08 | 4.00 ± 0.11 |
|  |  |  | 100 | 3.13 ± 0.02 | 3.56 ± 0.07 |
|  |  |  | 125 | 2.75 ± 0.16 | 3.21 ± 0.14 |
| CLE-AgNP |  |  | 25 | 1.51 ± 0.02 | 2.48 ± 0.30 |
|  |  |  | 50 | 1.17 ± 0.03 | 2.11 ± 0.03 |
|  |  |  | 75 | 0.55 ± 0.20 | 1.41 ± 0.02 |
|  |  |  | 100 | 0.48 ± 0.02 | 1.27 ± 0.03 |
|  |  |  | 125 | 0.43 ± 0.02 | 1.07 ± 0.03 |
| Genistein |  |  | 125 | 0.49 ± 0.02 | 1.33 ± 0.02 |

Parasitology Laboratory, Department of Zoology, Cooch Behar Panchanan Barma University, Cooch Behar, West Bengal, India* Corresponding author: [karpradip@gmail.com](mailto:karpradip@gmail.com)

Supplementary Table S1. In addition to the *in vitro* efficacy of CLE-AgNPs, as shown in Fig. 4, the *Raillietina* spp. parasites kept in control, and reference drug Genistein showed dose-dependent anthelmintic activity at all concentrations tested.
